# Maintaining progress for the most beautiful chart in the world

**DOI:** 10.1093/inthealth/ihz046

**Published:** 2019-01-01

**Authors:** Simon I Hay

**Affiliations:** Institute for Health Metrics and Evaluation, University of Washington, 2301 5th Avenue, Suite 600 Seattle, WA, USA

**Keywords:** child mortality, data visualization, global health, public health surveillance

## Abstract

The decline in child mortality over the past two decades has been described as the greatest story in global public health. Indeed, using modern tools and interventions, there has been remarkable progress, reducing deaths in children <5 y of age by nearly half from 2000 to 2017. However, as a consequence of persistent geographic inequalities, we fall short of the United Nations Sustainable Development Goal to end all preventable child deaths by 2030, with an estimated 44.6 million preventable deaths expected to occur by the target year. This article discusses how we might further improve the downward trend in child mortality over the next decade to end preventable child deaths.

In 1971, Ryan White, Justin Trudeau, Stella McCartney, Elon Musk, Tupac Shakur, Sara Seager and yours truly were born. Richard Nixon was Time Magazine’s ‘Person of the Year’. Intel released the world’s first microprocessor, Texas Instruments developed the first pocket calculator and Ray Tomlinson implemented the first internet-based e-mail. Also, an estimated 16.3 million children died before they reached the age of 5 y.^1^

It’s a sobering number. But when we look at Figure 1, we can see much has changed since 1971. Child mortality rates and the total number of child deaths in low- and middle-income countries (LMICs) have continuously trended downward.^1^ In 2015, Bill Gates tweeted the ‘most beautiful chart in the world’, which shows a decrease in deaths of children <5 y of age by more than half from 1990 to that year.^2^ He also revealed his favorite number—122 million—the number of children’s lives saved since 1990. These are children who would have died if mortality rates had stayed as they were at that time.^2^ Another heartening number is 375 million, which is the number of child deaths that were averted globally between 1971 and 2017 due to declining mortality rates.^1^

**Figure 1.**
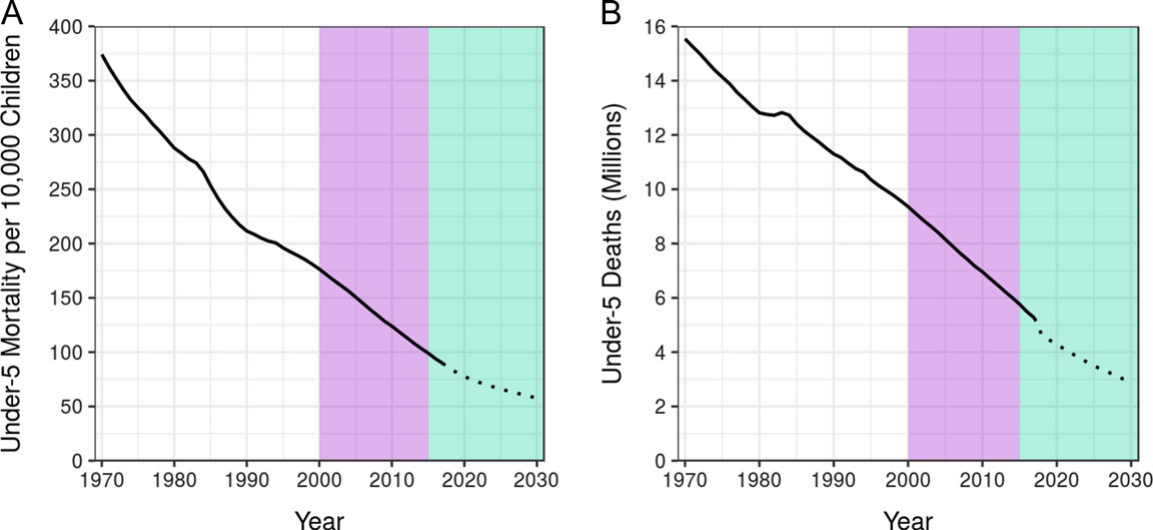
Trends in under-5 mortality in 103 LMICs, 1970–2030. (A) Under-5 mortality rate per 10 000 children. (B) Total under-5 deaths in millions. The mortality rate was determined by dividing the number of deaths by the population among all children <5 y of age. The purple area identifies the era of the MDGs and the green area represents the time remaining until 2030, the target year of the SDGs.^1^ The projected under-5 mortality rate and total deaths are indicated by the dotted portion of each line.^6^

When assessing child mortality, it is necessary to consider both the rate of these deaths (Figure 1A) and the total number of deaths (Figure 1B) for a given population, since population density varies enormously among locations. A preoccupation with child mortality rates can mask areas where the number of child deaths is high. In these areas, rates of child death may be comparatively low due to large population size, yet locations with high incidences of child death merit our attention for the simple reason that their toll is high in absolute terms. Certainly there is cause for optimism. The global level of mortality in children <5 y of age was down to 5.4 million deaths in 2017 while the global rate was 79.2 deaths per 10 000 children <5 y of age—a decrease of 57% from 1990.^1^ This remarkable progress made in reducing worldwide child mortality over the past two decades, using interventions such as immunization, safe water and sanitation, insecticide-treated nets, oral rehydration therapy and antibiotics, has been described as the greatest story in global public health.^3^

The global distribution of mortality in children < 5 y of age is obviously not uniform—97% of under-5 deaths occur in LMICs (Figure 2)^4^—and notable differences stand out when comparing total counts (Figure 2A) or rates of mortality (Figure 2B). Ethiopia, for example, had an under-5 mortality rate of 119.4 deaths per 10 000 children, compared with the sub-Saharan African average of 166.5 deaths per 10 000 children in 2017. However, its status as the second most populous country in sub-Saharan Africa means that the number of under-5 deaths there is high—198 942 children died there in 2017, an unacceptable number by anyone’s standard.

**Figure 2.**
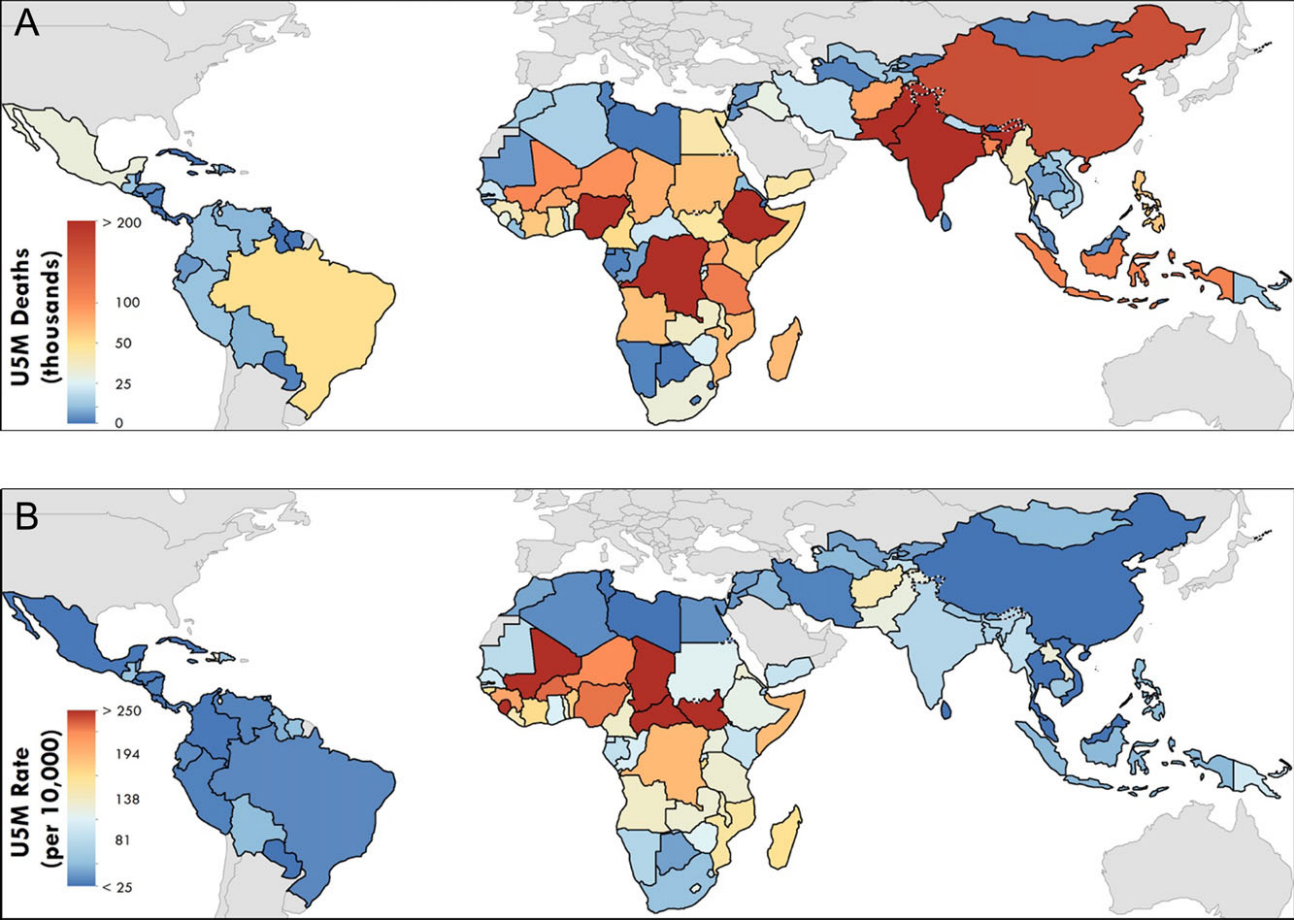
National-level estimates of under-5 mortality (U5M) in 103 LMICs in 2017. (A) Total number of deaths in children <5 y of age in 2017. (B) Probability of death for children <5 y of age in 2017.

The downward trends in both rates and counts affirm that we are on the right track. However, there is a glaring problem—the fact that both projected trends for 2030 fall well short of where they need to be.^5^ Despite the objective of the Millennium Development Goal (MDG) 4 to reduce under-5 mortality by two-thirds from 1990 to 2015, only 56 of 195 countries and territories worldwide have achieved an annualized rate of decline necessary to meet MDG 4; of these, only 11 are in Africa.^1^ If current trends continue, 44.6 million preventable child deaths will have occurred between now and the year 2030, when the era of the United Nations Sustainable Development Goals (SDGs) concludes.^6^ How do we improve on these trends so we successfully achieve the SDGs?

The Local Burden of Disease (LBD) project at the University of Washington’s Institute for Health Metrics and Evaluation uses geospatial estimation models to derive highly detailed estimates of levels and trends over long time series, which can be examined at scales as fine as 5×5 km (pixels), but more commonly, between second administrative units (e.g. provinces, districts, counties) within a country—the level at which policies can have the greatest impact. In a study published by the LBD project in 2017,^4^ precise geospatial modeling of under-5 and neonatal mortality at the 5×5-km scale across 46 African countries generated high-resolution estimates for all-cause mortality between 2000 and 2015. Not surprisingly, there was an overall decrease in under-5 mortality rates. However, many stark disparities were seen across the continent and within national borders, including areas needing to reduce their under-5 mortality rates by at least 8.8% per year to achieve the SDG 2030 target. These alarming subnational-level trends were masked by estimates summarized at the national level.

A more recent study used geostatistical tools to analyze childhood growth failure, illustrating the rates of stunting, wasting and underweight in children across Africa from 2000 to 2015.^7^ Similar methods were used to ascertain within-country inequalities in the educational attainment of women, which is linked to the health of mothers and their children.^8^ Also, diphtheria–pertussis–tetanus vaccine coverage and dropout were geospatially mapped for children 12–23 mo of age in 52 African countries.^9^ Despite the availability of low-cost, proven interventions, questions of where to direct investments so that they impact the greatest number of children persist. The insight into nutrition, education and vaccination provided by these studies can spur those responsible—policymakers, ministries, donors and agencies—to act increasingly appropriately and effectively.^10^

Using geospatial mapping to guide interventions to those areas most in need is part of a greater strategy sometimes described as precision public health.^11^ This is not to be confused with precision or personalized medicine, which focuses on individuals.^12^ It extends to populations, as in ‘providing the right intervention to the right population at the right time’.^13^ Determining how demographic, behavioral and social factors affect health is essential for the precise targeting of interventions.^14^ Dowell et al.^15^ highlighted four key tasks for precision public health: register births and deaths, track disease, incorporate laboratory analysis and train more people. Improved geospatial estimation and subsequent visualizations can aid in the second of these tasks, tracking geographic variability in levels and trends, and ultimately contribute to the policy aims of targeting and treating root causes.

In addition to tracking geographic inequalities, a greater impact could be made by mapping both proximal and distal determinants of child health. As described by Mosley and Chen’s^16^ classic analytical framework for childhood survival, distal determinants are societal, population or economic variables that can be used as indicators of welfare or disease but also measurably affect outcomes, while proximal determinants capture the specific disease processes leading to death. These drivers of health do not act in isolation, but rather interact as chains of events shaped by broader socioeconomic determinants. Past efforts, however, have not effectively leveraged what is known of both distal and proximal drivers; however, limiting opportunities to optimize intervention impacts on child vulnerability.

It is possible to identify both distal and proximate contributors to child mortality that can be mapped and are indicators used in global initiatives such as the SDGs. LBD is mapping such drivers as child welfare, including childhood growth failure and population levels of education mentioned previously, as well as malaria^17^ and diarrhoea.^18^ An understanding of the key distal and proximal determinants of overall child welfare mapped at a local resolution will enable us to more precisely determine where the most vulnerable children are, what is killing them and how we can most effectively intervene.

LBD has pinpointed how progress towards the MDGs has varied substantially at the subnational level (Figure 3A), revealing geographic inequalities in reducing the burden of child mortality and demonstrating an essential need to examine these trends with greater spatial resolution.^4^ These geographic inequalities have so far proved recalcitrant, with the highest mortality rates continuing to occur in much the same areas over decades. Despite overall progress, those who have been the worst off in terms of their relative exposure to child mortality—the ‘bottom 20%’—have largely remained so (Figure 3B).

**Figure 3.**
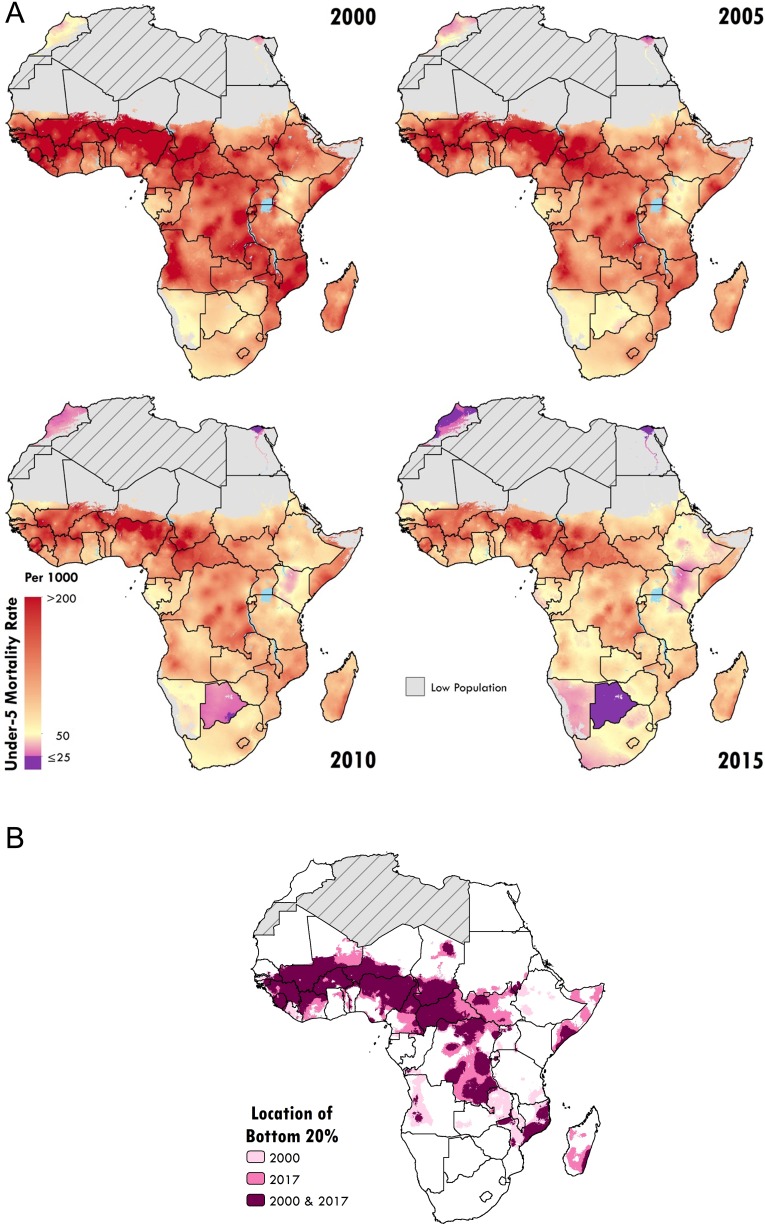
(A) Under-5 mortality rates at the 5×5-km resolution for 2000, 2005, 2010 and 2015. Pixels at the 5×5-km resolution are highlighted according to the under-5 mortality rate, from purple, indicating ≤25 deaths/1000 live births (the SDG 3.2 target), to orange, indicating >200 deaths/1000 live births. Grey indicates pixels with a population of fewer than 10 people. Grey areas with diagonal lines were not included in the analysis. (B) Locations with little to no improvements in the lowest 20% under-5 mortality rates across Africa between 2000 and 2017. The locations of the highest 20% of under-5 mortality rates, or the ‘bottom 20%’, are highlighted, indicating those with the highest likelihood of children dying before the age of 5 y in 2000 (pale pink) and 2017 (deep pink). The burgundy areas represent the aggregated data for 2000 and 2017, indicating where little to no change occurred during that time period. Grey areas with diagonal lines are not included in this analysis.

Admittedly, many of these locations are long thought of as being war-torn and poverty-stricken,^10^ arguably places where mapping determinants may not substantially contribute to progress. However, exemplar nations such as Senegal, Ethiopia and Uganda (Figure 3A) have transformed their under-5 mortality rates and absolute numbers of deaths.^5^ Ethiopia in particular has now achieved MDG 4 with an estimated under-5 mortality rate of 135.7 per 10 000 in the year 2015, a decline from 334.5 in 2000. This represents a reduction in mortality of 59%, compared with 44% for LMICs.^1^ At the subnational scale, I suspect there are many similar examples of positive change—exemplar regions, districts and communities—each with their own unique set of circumstances, offering myriad lessons to provide us with invaluable guidance as we move forward.

Researchers at the University of Global Health Equity (UGHE) have leveraged LBD’s local resolution maps of under-5 mortality to identify lessons learned from exemplar countries to inform future decision making. Specifically, LBD’s subnational analyses of rates in 2000 allowed the UGHE to examine Bangladesh, Cambodia, Nepal, Rwanda and Senegal. This work ultimately resulted in identifying and disseminating cross-cutting implementation strategies and policy lessons that can be adapted and adopted in other countries working to achieve similar progress.

Our international commitment is to put an end to all preventable child deaths by 2030, but sadly we may fail to reach this goal, even though certain locations have progressed rapidly. We cannot let the greatest story in public health lure us into an overly optimistic sense of achievement, leading us to forget the millions of marginalized children still dying each year. However, to quote Mr Gates again, ‘Being an optimist isn’t about knowing that life used to be worse. It’s about knowing how life can get better.’^19^ The potential of geospatial mapping to further bend the curve in child mortality downward can help make certain that the next decade tells the best story yet about ending preventable child deaths.
